# A pilot study to assess oral health literacy by comparing a word recognition and comprehension tool

**DOI:** 10.1186/1472-6831-14-135

**Published:** 2014-11-18

**Authors:** Khadija Khan, Brendan Ruby, Ruth S Goldblatt, Jean J Schensul, Susan Reisine

**Affiliations:** University of Connecticut School of Dental Medicine, Farmington, CT 06030 USA; Department of Craniofacial Sciences, Division of General Dentistry, University of Connecticut School of Dental Medicine, Farmington, CT 06030 USA; The Institute of Community Research, Hartford, CT 06106 USA; Division of Behavioral Sciences and Community Health MC3910, University of Connecticut School of Dental Medicine, Farmington, CT 06030 USA

**Keywords:** Older adults, Oral health, Health literacy, Oral health literacy, Rapid Estimate of Adult Literacy in Dentistry-30, Oral health knowledge, Gender, Oral health status, Comprehension, Word recognition

## Abstract

**Background:**

Oral health literacy is important to oral health outcomes. Very little has been established on comparing word recognition to comprehension in oral health literacy especially in older adults. Our goal was to compare methods to measure oral health literacy in older adults by using the Rapid Estimate of Literacy in Dentistry (REALD-30) tool including word recognition and comprehension and by assessing comprehension of a brochure about dry mouth.

**Methods:**

75 males and 75 females were recruited from the University of Connecticut Dental practice. Participants were English speakers and at least 50 years of age. They were asked to read the REALD-30 words out loud (word recognition) and then define them (comprehension). Each correctly-pronounced and defined word was scored 1 for total REALD-30 word recognition and REALD-30 comprehension scores of 0–30. Participants then read the National Institute of Dental and Craniofacial Research brochure “Dry Mouth” and answered three questions defining dry mouth, causes and treatment. Participants also completed a survey on dental behavior.

**Results:**

Participants scored higher on REALD-30 word recognition with a mean of 22.98 (SD = 5.1) compared to REALD-30 comprehension with a mean of 16.1 (SD = 4.3). The mean score on the brochure comprehension was 5.1 of a possible total of 7 (SD = 1.6). Pearson correlations demonstrated significant associations among the three measures. Multivariate regression showed that females and those with higher education had significantly higher scores on REALD-30 word-recognition, and dry mouth brochure questions. Being white was significantly related to higher REALD-30 recognition and comprehension scores but not to the scores on the brochure.

**Conclusions:**

This pilot study demonstrates the feasibility of using the REALD-30 and a brochure to assess literacy in a University setting among older adults. Participants had higher scores on the word recognition than on comprehension agreeing with other studies that recognition does not imply understanding.

## Background

The American Dental Association (ADA) defines oral health literacy as “the degree to which individuals have the capacity to obtain, process and understand basic health information and services needed to make appropriate oral health decisions” [1]. More health literate patients are better able to navigate the healthcare systems and have better health outcomes [2]. Those with low levels of health literacy are less likely to understand and follow treatment recommendations and lack the skills needed to make informed decisions about their own health care [3, 4]. Findings from the 2003 National Assessment of Adult Literacy (NAAL) showed that 71% of adults older than age 60 had difficulty in understanding print materials in prose format. This group also had the highest proportion of persons with health literacy defined as “below basic”, i.e. having no more than the most simple and concrete literacy skills such as signing forms, or locating easily identifiable information in a short, simple text [5].

Less is known about how health literacy impacts health-related outcomes for men and women. The NAAL in 2003 also reported that women in the United States had slightly higher overall levels of health literacy compared to men, and that a higher percentage of men than women had Below Basic Levels of Health Literacy.

While there is considerable literature on health literacy in medicine, health literacy in dentistry is a relatively new area of research. Several studies have shown that oral health literacy is associated with adults’ oral health status [6–8] as well as their children’s oral health [9–12]. Investigators suggest that oral health literacy may contribute to oral health disparities since those with low oral health literacy are more likely to be poor, not well educated, older and with limited English language skills [13]. Others suggest that those with low literacy are unable to communicate effectively with health care providers and this gap in communication may account for their worse oral health status [14, 15].

Dentists often encounter patients with limited oral health literacy skills in clinical practice, but they are not always able to identify those that may not be able to readily understand health explanations and instructions resulting in poor oral health outcomes. There is much need for quick, easy-to use oral health literacy tools that will allow for a comfortable experience for both providers and patients in identifying those that may need special methods of communication in clinical settings [16].

In dentistry, the most commonly used comprehension-based methods of assessing oral health literacy include the Test of Functional Health Literacy in Adults (TOHFLA) [17], the Test of Functional Health Literacy in Dentistry (TOHFLiD) [18] or the Oral Health Literacy Instrument (OHLI) [19]. These instruments are comprehension and numeracy tests which measure several aspects of health literacy, but take between 20–30 minutes to administer and are not suitable for use in a fast paced clinical care setting [17, 20].

Word-recognition tests such as the Rapid Estimate of Adult Literacy in Dentistry (REALD-30) have been developed to assess ability to read and pronounce common dental words correctly [21]. Thirty dental words are arranged in order of increasing difficulty. Richmond et al. developed a 99-item REALD with better predictive validity but the time taken to administer is much more than its increased predictive validity [20]. Therefore the 30-item REALD is more user-friendly and faster to use in a clinical setting.

While the REALD-30 is brief and easy to administer, it only measures recognition and pronunciation and does not assess understanding or comprehension. A recent review of health literacy instruments [22] found that the comprehension measures, such as the Medical Test of Functional Health Literacy in Adults (TOHFLA), were correlated with word-recognition tests such as the Rapid Estimate of Adult Literacy in Medicine (REALM) demonstrating that recognition is related to comprehension [17]. However, few studies have investigated the association between word recognition and comprehension in oral health literacy. Richman et al. [23] conducted a study of 44 parents of Head Start children to investigate how word recognition, vocabulary knowledge and comprehension of 35 pediatric dentistry terms were related. The authors found that word recognition was weakly correlated with comprehension and concluded that word recognition instruments are not a good indicator of oral health literacy. However, this study was limited by small sample size and its sole focus on pediatric dentistry. The REALD-30 has been used primarily in low income settings among young parents and has not been used extensively in other settings or with other populations. Very limited work has been done with oral health literacy among older adults. A recent study assessed the effectiveness of an educational intervention on oral health literacy and oral hygiene among older adults in assisted living complex. Oral health literacy was assessed using the REALD 30 and oral hygiene was assessed by the plaque index. REALD 30 scores were relatively high with mean scores at baseline of 23.21 (SD = 4.42) which improved significantly to 25.55 (SD = 4.6; p < 0.001). Oral hygiene also improved significantly, but was not related to improvements in the REALD 30 [24].

The primary objective of this study was to pilot test three methods to measure oral health literacy among older adults using the Rapid Estimate of Literacy in Dentistry (REALD-30) tool. We assessed word recognition and enhanced the REALD-30 tool by adding a comprehension component. We also assessed comprehension of a brochure about dry mouth. We compared these methods by investigating the associations among these methods and whether demographic characteristics differentially influence the oral health literacy assessments. We also aimed to assess the relationship between these health literacy tests and gender and oral health status and behaviors.

We propose that participants will obtain higher scores on the REALD-30 word recognition than on word comprehension and that the literacy test scores will be significantly correlated to each other. We also hypothesized that females, those that are younger, more educated and with more incomes will have higher literacy scores and that higher literacy scores would be associated with better oral health behaviors.

## Methods

### Sample and survey method

Participants were recruited from the waiting rooms of the dental clinics at the University of Connecticut Health Center. We used the methods described by Cohen [25] to estimate sample size and power for a two-tailed t-test with varying effect sizes, power at 0.80 and alpha level at 0.05. Cohen suggests that 0.5 is a medium effect size and 0.8 is a large size effect. A sample of 150 would be adequate to detect a medium effect size therefore we recruited 75 males and 75 females to assure we would be able to detect a moderate -large difference in literacy between males and females. To be eligible for participation participants had to be fluent English speakers and at least 50 years of age to have a large sample size and a broad range of adults to make comparisons within the groups of older adults. Informed consent was obtained from all participants. We explained the purpose and requirements of the study to each participant; informed them that participation was completely voluntary; that they could refuse to answer any questions or withdraw at any time; and that declining to participate would not affect their dental or other care at the University. The research protocol was approved by the University of Connecticut Health Center Institutional Review Board. Two hundred fifteen people were approached to participate in the study over a period of five weeks and approximately 30% declined to take part usually because they said they were in a hurry and said they did not have the time to do the survey.

Participation in the study included pronouncing words on the REALD-30 and explaining their meanings; reading a brochure about dry mouth and orally answering three comprehension questions about what they read; and completing a baseline survey that was also administered orally. Each participant took about 15–25 minutes to complete the process.

All interviews were conducted by two trained interviewers. The interviewers developed training materials which contained the data collection protocol, consent procedures and survey questions. The interviewers underwent five pretest interviews with non-participants to refine data collection methods and calibrate the interview protocol. The two interviewers also conducted 80 interviews together to ensure standardization of administration. No data were collected on inter-rater reliability. Participants received $5 in cash, a toothbrush and sample toothpaste for their participation.

### Oral health literacy assessments

#### REALD-30

Each participant was given a laminated copy of the REALD-30 list of words by the interviewer and asked first to read each word aloud and then to explain the meaning of the word. The REALD-30 was scored by assigning one point for each word correctly pronounced and one point for correctly explaining each word. Definitions for each word were developed from Google searches, dictionaries and other online resources. The interviewers decided whether the participant understood the meaning of the word based on this definition checklist. The REALD-30 words are either common dental terms, such as floss, that are easy to define or more technical terms, such as bruxism, that would have more precise definitions. Scores for word-recognition and word comprehension each ranged from 0 (lowest literacy) to 30 (highest literacy).

#### Dry mouth brochure

Participants were asked to read a brochure about Dry Mouth published by the National Institute of Dental and Craniofacial Research [26]. They could take as much time as they needed to but were not allowed to ask questions. The brochure, which is free to the public, is eight pages and discusses symptoms of dry mouth, the importance of saliva, the causes of dry mouth and potential treatments. The brochure has a Flesch-Kincaid reading level of Grade 5.4 and we picked it because it is easy to read and relevant to many older adults. After reading the brochure, participants were asked to answer orally three questions regarding the text and responses were scored according to Table 1. Total scores could range from 0–7.

**Table 1:** **Dry mouth comprehension assessment score**

Question	Possible points	Possible answers (from the brochure)
**1. What is dry mouth?**	0-1	Not enough saliva in the mouth
**2. What are three causes of dry mouth?**	0-3	Medicines, disease (Sjogrens etc.), radiation and chemotherapy, nerve damage
**3. List three ways how dry mouth can be treated?**	0-3	Consult doctor, change/adjust medications, medications, artificial saliva, sip water, sugarless candy/gum, avoid caffeinated drinks/alcohol/tobacco/spicy foods, use humidifier

Table 1 lists the three questions participants were asked to answer orally regarding the text, along with possible correct answers and how the answers were scored.

#### Participant characteristics

In addition to the literacy assessments, participants completed a survey containing questions about demographic characteristics, health status and behaviors.

#### Demographic characteristics

Data were collected on age in years, gender, race, educational attainment, marital status, and annual family income. Age was grouped into four categories: 50–55; 56–60; 61–69 and 70+ years. Gender was defined as male and female. Race was divided into white and non-white because of the limited number of participants other than white in the sample. Educational attainment was grouped into three categories: less than or equal to high school; some college and college degree or more. Marital status was grouped into married, separated, widowed or divorced, single, never married. Household income responses were organized into four groups: less than $10,000, $10-19,999, $20-$49,999 and greater than or equal to $50,000.

#### Health status/Health behaviors

Data on health status and health behaviors included self-rated overall health and oral health, brushing, flossing, dental visits, smoking cigarettes and dental insurance status. They reported number of times a day they brushed and flossed their teeth, when their last dental visit occurred, and whether they currently smoked.

### Data analysis

All analyses were conducted using IBM SPSS Statistics 20. Frequency distributions, means and standard deviations are presented where appropriate. Although the distributions of the REALD-30 scores were skewed, skewness statistics were within the acceptable level of −1.0 to +1.0 and thus employed both non-parametric and -parametric tests for the bivariate analysis using the Chi Square statistic and Pearson correlations. Values on the literacy assessments were categorized into quartiles for the chi square analyses. Linear regression was used in the multivariate analyses.

## Results

As shown in Table 2, a total of 150 participants comprising 75 males and 75 females participated in the study. The majority of the participants were patients at the Advanced Education in General Dentistry practice (60 percent) while the others were spouses, caregivers or friends accompanying the patients. About half of the participants were less than 60, although 20.6% were over the age of 70. Most participants had some college (40%) or had completed college (33.3%); most were white (80%), married or living with someone (43%), and had family incomes of less than $50,000 (64%). Participants rated their oral health worse than their overall general health. Those who rated *oral health* as fair/poor (41.3%) was twice as high than those who rated their *overall general health* as fair/poor (20.7%). Most participants reported they had visited the dentist in the past 6 months (71%). Most said they brushed at least twice a day (70%) and flossed at least once a day (45%) and few smoked (20%). Many had some sort of dental insurance in the form of either private insurance or Medicaid.

**Table 2:** **Descriptive characteristics of the participants**

Variable	
Demographic characteristics	Percent
**Age**	
50-55	22.7
56-60	24.7
61-69	32.0
70+	20.6
**Gender**	
Male	50
Female	50
**Education**	
≤High School	26.7
Some college	40.0
College +	33.3
**Race**	
White	80.0
Non-White	20.0
**Marital status**	
Married	42.7
Sep/Wid/Div	39.3
Single	18.0
**Income**	
<$10,000	20.0
$10-19,999	22.0
$20-$50,000	22.07
≥$50,000	27.3
Refused	8.0
**Health status/health behaviors**	
**Overall health**	
Excellent/Very Good	42.0
Good	37.3
Fair/Poor	20.7
**Oral health rating**	
Excellent/Very Good	24.0
Good	34.7
Fair/Poor	41.3
**Last dental visit**	
≤6 months	70.7
>6 months	29.3
**Tooth brushing**	
≤Once a day	30.0
≥Twice a day	70.0
**Floss**	
< Once a day	51.1
≥ Once a day	48.9
**Have dent insurance**	
Yes	55.3
No	44.7
**REALD WR – Quartiles**	
1-19	26.0
20-24	28.0
25-27	22.0
28-30	24.0
**REALD WC – Quartiles**	
1–13	30.0
14–15	20.7
16–19	25.3
20-30	24.0
**Total score (0–7)**	
0, 1, 2,3, 4	32.0
5	21.3
6	28.0
7	18.7

Table 3 displays the word list and the percent correct on recognition and comprehension. *Sugar, Smoking* and *Brush* were the only three words that all participants could both pronounce and define correctly, although almost all participants could pronounce and define *Floss, Braces, Extraction and Denture*. In contrast, *Hypoplasia, Fistula, Temporomandibular, Hyperemia* and *Apicoectonomy* were more difficult words with 50 percent or less of the participants correctly pronouncing these words. Less than 25 percent of the participants could correctly define *Bruxism, Dentition, Gingiva, Incipient, Hypoplasia, Fistula, Temporomandibular, Hyperemia and Apicoectonomy.* Some of the limitations in the word recognition approach to literacy assessment are apparent in Table 3 which illustrates that participants could pronounce words but could not define them. These included, for example, caries, periodontal and sealant. Internal reliability for both scales was very positive. Chronbach’s alpha for the REALD-30 WR was 0.902 and for REALD-30 COMP alpha was 0.851.

**Table 3:** **Percent correct on each REALD 30 word recognition and comprehension**

REALD 30 words	% Correct	
	Recognition	Comprehension
1. Sugar	100%	100%
2. Smoking	100	100
3. Floss	98.7	99.3
4. Brush	100	100
5. Pulp	96.7	61.6
6. Fluoride	96.0	86.1
7. Braces	99.3	98.6
8. Genetics	94.7	81.5
9. Restoration	98.0	66.9
10. Bruxism	55.0	10.6
11. Abscess	97.4	94.0
12. Extraction	98.0	99.3
13. Denture	98.0	98.0
14. Enamel	97.4	91.4
15. Dentition	66.2	5.3
16. Plaque	97.4	91.4
17. Gingiva	91.4	53.6
18. Malocclusion	57.0	28.5
19. Incipient	64.9	13.2
20. Caries	81.5	28.5
21. Periodontal	86.1	36.4
22. Sealant	93.4	32.5
23. Hypoplasia	39.1	0
24. Halitosis	76.8	70.9
25. Analgesia	61.6	41.7
26. Cellulitis	68.2	20.5
27. Fistula	47.0	18.5
28. Temporomandibular	28.5	17.9
29. Hyperemia	37.7	6.0
30. Apicoectomy	6.6	3.3
**Literacy scores**	**Mean (sd)**	
**Word recognition**	22.98 (5.1)	
**Comprehension**	16.05 (4.3)	

**Figure 1:**
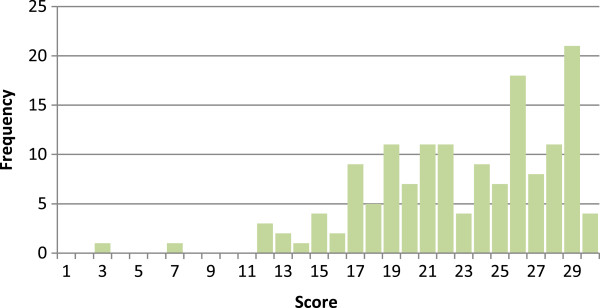
**Range of scores for Word-Recognition.** Mean score: 22.98 ±5.1, Median score: 24.

**Figure 2:**
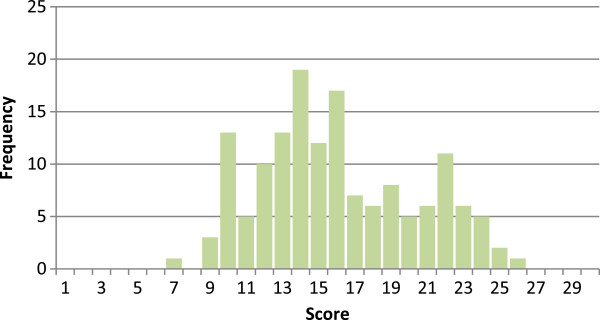
**Range of scores for REALD- comprehension.** Mean score: 16.05 ± 4.3, Median score: 15.

Figure 1 shows the distribution of the REALD-Word Recognition (REALD-WR) scores which ranged from 3–30 with a mean of 22.98 (SD = 5.1). Figure 2 presents the distribution of the REALD-Comprehension (REALD-COMP) with a range of 7–26 and mean of 16.1 (SD = 4.3). It is also apparent from the two figures that the distribution of the scores is lower for comprehension. Participants scored significantly higher on word recognition compared to comprehension (p < 0.001).The data provide support for the first hypothesis that word recognition scores are higher than comprehension scores. Results for the dry mouth brochure are shown in Table 4. Most people could define dry mouth after reading the brochure. Most participants also could list at least one cause and one treatment of dry mouth, although participants tended to have higher scores on reporting treatments for dry mouth (mean = 2.3; SD = 0.86) compared to causes of dry mouth (mean =1.9; SD = 0.90). Overall, scores for the dry mouth brochure comprehension, which could range from 0 to 7, were relatively high, with a mean score of 5.1 (SD = 1.60) and median of 5 for the sample.

**Table 4:** **Frequency and mean scores (sd) for dry mouth brochure items**

	Frequency - percent	Mean score (sd)
**Dry mouth brochure items**		
**Definition of dry mouth correct**	90.7%	(N/A)
**Causes of dry mouth (0–3)**		1. 9 (0.90)
0	7.3	
1	24.0	
2	40.7	
3	28.0	
**Treatments for dry mouth (0–3)**		2.3 (0.86)
0	4.0	
1	15.3	
2	30.7	
3	50.0	
**Total score - Quartiles**		5.1 (1.6)
0, 1, 2, 3, 4	32.0	
5	21.3	
6	28.0	
7	18.7	

Tables 5 and 6 show the bivariate analyses of participant characteristics and literacy assessment. Scores for REALD-30 word recognition and comprehension were divided into quartiles. Quartiles for REALD-30 recognition were 1–19, 20–24, 25–27 and 28–30; for REALD- 30 comprehension, quartiles were 0–13, 14–15, 16–19 and 20–30. Gender, education, race and tooth brushing frequency were significantly associated with scores on both recognition and comprehension on the REALD-30. Males, those with high school or less education, non-whites and those who brushed less than once per day more likely to have low scores on both measures compared to females, those with more education, whites and those who brushed more frequently. For dry mouth brochure comprehension scores shown in Table 7, gender, education, marital status and income were significantly related to older scores. Males, those with less than a high school education, non-white, and those with an income of $20,000 were more likely to have lower dry mouth comprehension scores. Pearson correlations among the three measures are presented in Table 8. REALD-30 recognition and comprehension are significantly and highly correlated (r = 0.779; p <0.01). Comprehension of the brochure also is significantly correlated with scores on both REALD-30 measures.

**Table 5:** **Demographic and health status/Behavior characteristics and quartile scores on REALD-30 word recognition**

	1-19	20-24	25-27	28-30
Demographics	Percent			
**Age**				
50-55	32.4	32.4	20.6	14.7
56-60	32.4	27.0	16.2	24.3
61-69	16.7	22.9	27.1	33.3
70+	25.8	32.3	22.2	19.4
Gender*				
Male	30.7	32.0	24.0	13.3
Female	21.3	24.0	20.0	34.7
Education***				
≤HS	60.0	22.5	17.5	0.0
Some college	18.3	33.3	18.3	30.0
College +	8.0	26.0	30.0	36.0
Race***				
White	18.3	27.8	26.1	27.8
Non-White	51.4	28.6	8.6	11.4
**Marital status**				
Married	21.9	23.4	26.6	28.1
Not Married	29.1	31.4	18.6	20.9
**Income (n = 137)**				
<$10,000	40.0	33.3	13.3	13.3
$10-19,999	39.4	27.3	18.2	15.2
$20-$50,000	9.1	30.3	24.2	36.4
≥$50,000	24.4	22.0	24.4	29.3
**Health status/health behaviors**				
**Overall health**				
Excellent/Very Good	15.9	25.4	30.2	28.6
Good	28.6	30.4	16.1	25.0
Fair/Poor	41.9	29.0	16.1	12.9
**Oral health rating**				
Excel/VG	19.4	13.9	30.6	36.1
Good	23.1	30.8	26.9	19.2
Fair/Poor	32.3	33.9	12.9	21.0
**Last dental visit**				
≤6 months	25.0	31.8	15.9	27.3
>6 months	26.4	26.4	24.5	22.6
Tooth brushing*				
≤Once/day	47.7	17.8	15.6	20.0
≥Twice/day	22.9	21.9	29.5	25.7
**Floss**				
<Once/day	32.4	25.4	21.1	42.9
≥Once/ day	16.2	32.4	22.1	29.4
**Have dent insurance**				
Yes	32.5	22.9	20.5	55.6
No	17.9	34.3	23.9	23.9
**Literacy measures**				
REALD WC – Quartiles***				
1–13	62.2	31.1	6.7	0.0
14–15	22.6	54.8	19.4	3.2
16–19	10.5	23.7	42.1	23.7
20-30	0.0	5.6	22.2	72.2
Total score – Quartiles***				
**0, 1, 2, 3, 4**	45.8	35.4	12.5	6.3
**5**	31.3	28.1	18.8	21.9
**6**	14.3	33.3	33.3	30.6
**7**	3.6	7.1	35.7	

*p < 0.05; **p < 0.01; ***p < 0.001.

**Table 6:** **Demographic and health status/Behavior characteristics and quartile scores on REALD-30 word comprehension**

Demographics	1-13	14-15	16-19	20-30
**Age**				
50-55	32.4	32.4	17.6	17.6
56-60	32.4	21.6	27.0	18.9
61-69	16.7	14.6	31.3	37.5
70+	45.2	16.1	22.6	16.1
Gender**				
Male	41.3	24.0	21.3	13.3
Female	18.7	17.3	29.3	34.7
Education***				
≤HS	67.5	17.5	12.5	2.5
Some college	23.3	20.0	28.3	28.3
College +	8.0	24.0	32.0	36.0
Race*				
White	25.2	18.3	28.7	27.8
Non-White	45.7	28.6	14.3	11.4
**Marital status**				
Married	23.4	18.8	32.8	25.0
Not Married	34.9	22.1	19.8	23.3
**Income (n = 137)**				
<$10,000	40.0	26.7	16.7	16.7
$10-19,999	45.5	18.2	18.2	18.2
$20-$50,000	18.2	15.2	30.3	36.4
≥$50,000	19.5	19.5	39.0	22.0
**Health status/health behaviors**				
**Overall health**				
Excellent/Very Good	25.4	17.5	28.6	28.6
Good	30.4	23.2	19.6	26.8
Fair/Poor	38.7	22.6	29.0	9.7
**Oral health rating**				
Excel/VG	25.0	16.7	30.6	27.8
Good	26.9	19.2	32.7	21.2
Fair/Poor	35.5	24.2	16.1	24.2
**Last dental visit**				
≤6 months	29.2	21.7	24.5	24.5
>6 months	31.8	18.2	27.3	22.7
Tooth brushing*				
≤Once/day	46.7	17.8	15.6	20.0
≥Twice/day	22.9	21.9	29.5	25.7
**Floss**				
<Once/day	35.2	19.7	50.0	18.3
≥Once/ day	19.1	23.5	27.9	29.4
**Have dent insurance**				
Yes	28.9	20.5	28.9	21.7
No	31.3	20.9	20.9	26.9
**Literacy measure**				
Total score – Quartiles***				
**0, 1, 2, 3, 4**	58.3	29.2	10.4	2.1
**5**	34.4	28.1	15.6	21.9
**6**	11.9	19.0	40.5	28.6
**7**	2.2	0	39.3	57.1

*p < 0.05; **p < 0.01; ***p < 0.001.

**Table 7:** **Demographic and health status/Behavior characteristics and quartile scores on dry mouth brochure**

Demographics	0-4	5	6	7
**Age**				
50-55	29.4	29.4	29.4	11.8
56-60	29.7	27.0	24.3	18.9
61-69	20.8	16.7	37.5	25.0
70+	54.8	12.9	16.1	16.1
Gender*				
Male	40.0	25.3	20.0	14.7
Female	24.0	17.3	36.0	22.7
Education***				
≤HS	57.5	22.5	20.0	0
Some college	23.3	26.7	31.7	18.3
College +	22.0	14.0	30.0	34.0
**Race**				
White	29.6	20.0	28.7	20.9
Non-White	40.0	22.9	25.7	11.4
Marital status*				
Married	20.3	29.7	32.8	17.2
Not Married	40.7	15.1	24.4	19.8
Income (n = 137)*				
<$10,000	46.1	20.0	16.7	16.7
$10-19,999	48.5	18.2	18.2	15.2
$20-$50,000	21.2	18.2	30.3	30.3
≥$50,000	14.6	29.3	41.5	14.6
**Health status/health behaviors**				
**Overall health**				
Excellent/Very Good	25.4	22.2	34.9	17.5
Good	30.4	21.4	26.8	21.4
Fair/Poor	48.4	19.4	16.1	16.1
**Oral health rating**				
Excel/VG	25.0	19.4	38.9	16.7
Good	32.7	21.2	28.8	17.3
Fair/Poor	35.5	22.6	21.0	21.0
**Last dental visit**				
≤6 months	35.8	21.7	27.4	22.9
>6 months	22.7	20.5	29.5	27.3
**Tooth brushing**				
≤Once/day	42.2	26.7	22.2	8.9
≥Twice/day	27.6	19.0	30.5	22.9
**Floss**				
<Once/day	28.2	21.1	28.2	22.5
≥Once/ day	32.4	23.5	27.9	16.2
**Have dent insurance**				
Yes	32.5	21.7	32.5	13.3
No	31.3	20.9	22.4	25.4

*p < 0.05; **p < 0.01; ***p < 0.001.

**Table 8:** **Pearson correlations among REALD-30 word recognition, REALD-30 word comprehension and dry mouth brochure scores**

Measure	REALD-30 word comprehension	Dry mouth brochure
**REALD-30 word recognition**	0.779**	0.554**
**REALD-30 word comprehension**	--	0.628**

*p < 0.05; **p < 0.01; ***p < 0.001.

The multivariate regression analyses for REALD-30 recognition, REALD-30 comprehension and the dry mouth brochure are shown in Table 9. All three regression models are highly significant (p < 0.001). Three variables were significantly related to the REALD-30 measures: females, those with higher educations and whites had higher scores on both measures compared to males, those with less than a high school education and non-whites. It is noteworthy that for REALD-30 comprehension analysis, gender had a higher significance level and larger regression coefficient than for recognition, suggesting that gender may be more important for comprehension in this sample. Results of the multivariate analysis of the dry mouth scores were similar to the results for REALD-30 except that race did not have a significant effect on comprehensions.

**Table 9:** **Result of the multiple regression analyses with REALD-30 Word Recognition, REALD-30 Word Comprehension and Dry Mouth Brochure Scores as dependent measures**

Variable	Standardized regression coefficients		
	REALD-30 word recognition	REALD-30 word comprehension	Dry mouth brochure
**Demographic Characteristics**			
**Age**	−0.004	−0.068	−0.102
**Gender**	0.153*	0.333***	0.177*
**Education**	0.368***	0.423***	0.347***
**Race**	0.293***	0.220**	0.107
**Marital status**	−0.017	−0.002	0.067
**Income**	0.048	−0.003	0.026
**Health status/behaviors**			
**Oral health rating**	0.081	0.080	0.048
**General health Rating**	0.120	0.015	0.036
**Tooth brushing**	0.133	0.039	0.148
**Flossing**	0.022	0.043	−0.129
**Dental visit in past 6 months**	−0.022	−0.017	−0.074
**Adjusted R** ^**2**^	0.367	0.363	0.211
**F (df)**	8.86 (11;138) p < 0.001	8.71 (11;138) p < 0.001	4.62 (11;138) p < 0.001

*p < 0.05; **p < 0.01; ***p < 0.001.

## Discussion

This study builds on the previous work of Lee and colleagues [21] developing a brief measure of oral health literacy based on word recognition by adding a comprehension component to the REALD-30. As with other studies that use the REALD-30, our participants had relatively high scores (mean 22.98; SD = 5.1) on word recognition and the distribution was skewed towards higher scores. The distribution on the comprehension scores was less skewed than the word recognition scores and the sample had somewhat lower scores (mean =16.05; SD = 4.3). REALD-30 comprehension may be a better tool to differentiate among literacy levels. The dry mouth brochure scores also were high with a highly skewed distribution. This result may be due to the 5th grade reading level of the brochure making it more accessible to those with lower literacy ability.

Several authors [23, 27] have discussed the limitations of the word recognition approach to literacy assessment because word recognition does not necessarily indicate that the person understands the meaning of the word. The findings from this study support this assertion in that we found that scores on the REALD word recognition measure were higher than word comprehension measure indicating that participants were able to pronounce more words than they could define. Often participants correctly pronounced words for which they did not know dental meanings such as *pulp* which they often associated with orange juice pulp or *gingiva* which many defined as gum disease because of their familiarity with *gingivitis*. That being said, REALD-30 scores on recognition and comprehension were highly correlated suggesting that REALD-30 word recognition may be useful as a screening measure of oral health literacy when time is limited in the clinical context or as an initial assessment for further follow-up. REALD-30 word-recognition and comprehension scores were also correlated with measures of comprehension of the dry mouth brochure lending additional evidence for the validity of the REALD-30 measures as oral health literacy assessment tools.

A secondary focus of this paper was to assess gender differences in oral health literacy. The effect of gender was highly significant at the bivariate and multivariate levels with females demonstrating greater oral health literacy on all measures. The effects of gender on oral health literacy has not been investigated extensively because much of the development of the REALD-30 has been done with young females [10, 11, 28] without emphasis on the analysis of gender. An early study of oral health literacy among dental patients in private practice (n = 101) which included both males and females did not find gender differences in the REALD-30 word recognition scores [29]. The participants in our study consisted of older adults (ages 50 and older) who were dental patients at a University dental practice. Oral health literacy in our sample was similar to Jones et al’s [29] sample in the dental practice which had a mean score on the REALD-30 of 23.9 (SD = 1.3). Our scores also similar to Hjertstedt et al., [24] who reported baseline scores on the REALD-30 of 23.21 (SD = 4.42) although their sample was relatively small (n = 67). Our participants had considerably higher mean scores on word recognition using the REALD-30 compared to samples of young, low income mothers (mean score of 15.8; SD = 5.1; [11]).

The bivariate and multivariate analyses provide limited support for our expectations about the REALD 30 and participant characteristrics. As expected, education was consistently related to health literacy measures. Race was significantly related to word recognition and comprehension using the REALD-30 but not to literacy assessed by the dry mouth brochure. Contrary to our expectations, self –reported oral health status was not significantly related to any of the three literacy assessments although tooth brushing was related to word recognition at the bivariate level.

There are two words in the REALD-30 floss and extraction that have higher scores on REALD-30 comprehension that word-recognition. This occurred because some adults had difficulty pronouncing certain words but could still define that same word correctly. Hence the participant received a point for comprehension but not recognition. It is possible that the participant could read the word but could not pronounce it because of language difficulties. This is another example of where word-recognition itself is limited because it measures correct pronunciation and not understanding of the word.

Our study also demonstrated that the dry mouth brochure is a useful and practical method of measuring oral health literacy. Participants found it easy and quick to read, taking an average of about 3 minutes to complete. Participants’ comprehension scores on the brochure were quite high suggesting that this tool is accessible to those with lower literacy. Overall we suggest that the dry mouth brochure is a comprehensive, multi-faceted approach to measuring literacy because it allows patients to read the brochure, synthesize the information they read and then recall it during questions asked of them. Use of such brochures to measure health literacy could have many uses in clinical practice such as in patients with high caries risk during oral hygiene instruction.

Our results are somewhat consistent with other studies in finding a relatively high level of oral health literacy measured by word recognition among adult dental patients. As with other studies, we found significant associations between oral health literacy and education, but did not find a relationship between oral health literacy, self-rated oral health or oral health behaviors. Our findings may be related to our focus on older adults or on dental patients who have been treated at a university dental practice. However, our study demonstrates the feasibility and acceptability of using the REALD-30 (both word-recognition and comprehension) in dental treatment setting among older adults.

### Limitations

There are several limitations to the study. We employed a sample of convenience and recruited half males and half females to assure that we had adequate numbers to assess the effects of gender. The results therefore have limited generalizability. Our participants included patients for scheduled dental treatment, regular follow-up and emergency walk-in care. We did not differentiate among these types of patients which could have affected the results.

Evaluating comprehension of the REALD-30 words was somewhat subjective. We developed a list of standard definitions for each term on the REALD-30 and scored comprehension according to the definitions on our list based on the interviewers’ judgment on participants’ understanding of the terms. Further work is needed to standardize acceptable definitions for each of the terms in the REALD-30. An example would be using a multiple choice answer for each REALD-30 world to quantify comprehension more objectively although this may not be feasible for a quick, rapid oral health literacy test.

Our study is also limited to English speakers because this version of the REALD-30 is currently only validated in English. Many of our patients in the dental clinic who speak Spanish or other languages are not accounted for in the study because they were not eligible to participate. Therefore more work is needed to develop oral health literacy tools in Spanish as is currently being done by Lee et al. [30].

## Conclusion

In this study we developed two oral health literacy comprehension tools, the REALD-30 comprehension and Dry Mouth Brochure comprehension, both of which correlated with the REALD-30 word-recognition test previously developed by Lee et al. [21]. Our study shows that using brief comprehension tests along with word-recognition tests enhance oral health literacy measurements, as adults may recognize words but may not actually understand them. Such literacy tests have use both in clinical practice and research settings. However further development of these comprehension tools is needed to make them more effective for use in clinical practice.

## Abbreviations

NAAL: National Assessment of Adult Literacy
OHLI: Oral Health Literacy Instrument
REALD-30: Rapid Estimate of Adult Literacy in Dentistry −30
REALD-WC: Rapid Estimate of Adult Literacy in Dentistry Word Comprehension
REALD-WR: Rapid Estimate of Adult Literacy in Dentistry Word Recognition
REALM: Rapid Estimate of Adult Literacy in Medicine
TOHFLA: Test of Functional Health Literacy in Adults
TOHFLiD: Test of Functional Health Literacy in Dentistry.
